# Anxiety Levels Among Cancer Patients: Age-Related Differences

**DOI:** 10.1192/j.eurpsy.2026.11230

**Published:** 2026-07-31

**Authors:** B. Purev, M. Dashdondog, P. Sarantuya, T. Mytav, J. Erdenebat, S. Surlegbaatar, B. Ganzorig, U. Enkhtur

**Affiliations:** 1 Department of Medical Science, Etugen University; 2 Amjilt Cyber School, Ulaanbaatar, Mongolia

## Abstract

**Introduction:**

Globally, 301 million people suffer from anxiety disorders. In Mongolia, the incidence of cancer has been increasing annually in recent years. The country ranks among the highest worldwide in cancer-related mortality, with liver cancer ranking first in both incidence and mortality, and lung and cervical cancers ranking third in mortality. According to the National Cancer Center of Mongolia, 6,885 new cancer cases were registered nationwide in 2022. According to the World Health Organization (WHO), in 2020, 19.3 million people were diagnosed with cancer 9.9 million people died from the disease. By 2040, new cancer cases are expected to double in Africa and increase 1.6 times in Asia, with cancer-related deaths projected to reach 28.9 million globally. During the COVID-19 pandemic, the prevalence of anxiety disorders increased by 26% within a year. In Mongolia, liver cancer accounts for 33.2% of all newly diagnosed cancers, gastric cancer 17.2%, lung and bronchial cancer 7.6%, esophageal cancer 5%, and cervical cancer 4.6%.

**Objectives:**

This study aims to examine whether anxiety differs among cancer patients across different age groups.

**Methods:**

Data for the study were collected from cancer patients using a total of 14 questions, including the GAD-7 scale developed by the World Health Organization (WHO) for use by primary healthcare providers, and 7 general demographic questions.

**Results:**

Of the 120 participants, 65.8% were female and 34.2% were male. By age group, 15.8% (n=19) were aged 25 years and under, 22.5% (n=27) were aged 26–35, 20% (n=24) were aged 36–45, 21.7% (n=26) were aged 46–55, 13.3% (n=16) were aged 56–65, and 6.7% (n=8) were aged 66 years and older. The majority of participants (64.2%) were between the ages of 26 and 55. Mild anxiety was observed in 46 participants (47.9%), while moderate anxiety was found in 34 participants (35.4%). Statistical analysis of the relationship between anxiety and age among cancer patients revealed a statistically significant difference in participants aged 25 and under (P = .057).

**Conclusions:**

A total of 120 participants were included in the study. Anxiety levels were higher among younger participants, and significant differences were observed between age groups.

**Disclosure of Interest:**

None Declared

